# Developing a Low-Cost Force Treadmill via Dynamic Modeling

**DOI:** 10.1155/2017/9875471

**Published:** 2017-06-04

**Authors:** Chih-Yuan Hong, Lan-Yuen Guo, Rong Song, Mark L. Nagurka, Jia-Li Sung, Chen-Wen Yen

**Affiliations:** ^1^Department of Mechanical and Electromechanical Engineering, National Sun Yat-sen University, Kaohsiung, Taiwan; ^2^Department of Sports Medicine, Kaohsiung Medical University, Kaohsiung, Taiwan; ^3^School of Engineering, Sun Yat-sen University, Guangzhou, China; ^4^Department of Mechanical Engineering, Marquette University, Milwaukee, WI, USA; ^5^Department of Physical Therapy, Kaohsiung Medical University, Kaohsiung, Taiwan

## Abstract

By incorporating force transducers into treadmills, force platform-instrumented treadmills (commonly called force treadmills) can collect large amounts of gait data and enable the ground reaction force (GRF) to be calculated. However, the high cost of force treadmills has limited their adoption. This paper proposes a low-cost force treadmill system with force sensors installed underneath a standard exercise treadmill. It identifies and compensates for the force transmission dynamics from the actual GRF applied on the treadmill track surface to the force transmitted to the force sensors underneath the treadmill body. This study also proposes a testing procedure to assess the GRF measurement accuracy of force treadmills. Using this procedure in estimating the GRF of “walk-on-the-spot motion,” it was found that the total harmonic distortion of the tested force treadmill system was about 1.69%, demonstrating the effectiveness of the approach.

## 1. Introduction

In walking and running, the inertial force acting on the human body is equal to the sum of the ground reaction force (GRF) exerted by the ground on the foot and the gravitational force of the body weight. Many important gait parameters can be derived from the GRF. These include temporal features such as the time instants of heel strike and toe off and the time durations of stance and swing phases as well as the step frequency. As a result, GRF can provide important information about gait behavior.

GRF data have been used to investigate gait symmetry [1], calculate leg stiffness [2], quantify impacts [3], understand propulsion and braking [4], compute muscle forces, joint forces and moments [5, 6], explain running economy [7, 8], and top running speeds [9]. GRF data have also been used to assess the effects of health-related conditions that can influence gait. These conditions include knee replacement [10], hip arthroplasty [11], aging effect [12], knee arthrosis [13], Parkinson's disease [14], peripheral arterial disease [15, 16], patellofemoral pain syndrome [17], osteoarthritis [18], cerebral palsy [19], multiple sclerosis [20], lower extremity muscle fatigue [21], stroke [22, 23], weighted walking [24], and hemiplegia [25].

To measure GRF during gait, most previous studies have relied on a force platform-embedded walkway. The most common configuration of a force platform consists of a metal plate mounted on load cells that give an electrical output proportional to the force applied to the plate. Typically, only a few steps of gait data are collected in each experimental trial. The necessity of proper foot placement on the force platform also complicates the experimental process. In addition, intentional behavior is likely to change the GRF and alter the gait pattern. This problem is particularly pronounced in testing individuals who exhibit gait difficulty. It is very difficult to perform constant speed walking or running studies using floor-mounted force platforms.

Since it has been found that the differences between treadmill and overground locomotion are small [26–28] and can be negligible after only a few minutes of treadmill-walking practice [29], treadmills have been employed extensively to study gait. To enhance the utility of treadmills, force platform-instrumented treadmills (commonly called force treadmills) have been used to quickly and continuously collect large amounts of GRF data during gait. These force treadmills offer several advantages over conventional walkway-based measurement systems. First, force treadmills reduce the time and space requirements substantially. Second, with a treadmill, controlling the speed of locomotion becomes a straightforward task. Third, body weight support modules can be added to the treadmills to ensure safety. Fourth, it is easier to integrate complementary measurement devices (such as electromyographic systems and oxygen consumption-measuring instruments) in the treadmill design in comparison to using a walkway-based system.

Treadmill training is frequently prescribed as a treatment option for patients with gait abnormalities. By using a force treadmill to quantitatively analyze gait patterns and detect gait abnormalities, medical therapists can adjust the intensity of treadmill training on an individual basis. In addition, previous studies have shown that the feedback of auditory, vibrotactile, and visual gait information can alter or improve gait features such as walking speed [30, 31], gait coordination [32], trunk sway [33], stride length [31], hip mechanics [34], cadence [31], step length symmetry [35], knee movement [36], gait cycle length [37], duration of gait [37], and swing phase speed [37]. With the capability of generating many important gait features, force treadmills represent an ideal platform for implementing such biofeedback systems.

Based on the location of the force transducers, force treadmills can be divided into two categories: direct measurement force treadmills (DMFTs) and indirect measurement force treadmills (IMFTs). By incorporating force platforms internally, DMFTs can measure GRF directly without considering the structural dynamics of the treadmill body [38–42]. Typically, DMFTs were built by installing force platforms under the track surface of the treadmill. This conceptually simple setup, however, requires complex mechanical design and a tedious assembly and calibration process in order to prevent erroneous force components generated by the moving parts (the motor and mechanism) of the treadmill [39, 42–44].

In contrast, by mounting the treadmill on top of force transducers, IMFTs simplify the mechanical design of force treadmills [45–49]. The friction forces generated by the moving components (such as belt, motor, and rollers) of the treadmill become internal forces and are not measured by the force sensors attached externally to the treadmill frame. The tradeoff of such a simplified design is the potential infidelity of the GRF measurements. Unless the treadmill frame can be made rigid, forces transmitted to the force transducers of the IMFT are generally not the same as the actual GRF applied to the treadmill track surface. To resolve this problem, current IMFTs are designed to possess a very high natural frequency to prevent the GRF from exciting the dynamics of the treadmill structure. This high structural natural frequency specification can only be achieved when the treadmill body is light and rigid. As a result, one needs to use low-density, high-stiffness materials in a specially designed mechanical structure for the treadmill frame. These requirements inevitably increase the manufacturing cost. The other reason for the high price of current force treadmill systems is that, due to their special design requirements, these treadmills are typically custom made or manufactured in very small quantities. In comparison, standard exercise treadmills are mass produced and, as such, much more affordable.

Considering the utility of force treadmills and the fact that their high cost has limited their adoption, the goal of this study is to introduce a systematic approach to convert a standard exercise treadmill into a force treadmill via a straightforward system identification method. A distinct feature of the proposed approach is that it relaxes the high structural natural frequency requirement for the treadmill frame. As a result, the construction cost of the force treadmills can be reduced considerably. This work also proposes an experimental procedure to assess the GRF measurement accuracy of force treadmills.

## 2. Methods

### 2.1. The Dynamic Modeling Method

This subsection identifies the dynamic specifications that need to be satisfied by conventional IMFTs. Furthermore, it introduces the basic idea of the proposed approach by addressing the problems caused by such specifications. Denoting the force applied to the IMFT track surface as *x*(*t*) and the force transmitted to the force transducers placed under the IMFT body as *y*(*t*), this study assumes that the GRF transmission dynamics of transmitting the force from *x*(*t*) to *y*(*t*) can be modeled as a linear time-invariant single-input single-output (SISO) system. In particular, with *x*(*t*) as the input and *y*(*t*) as the output, the GRF transmission dynamics of the IMFT are represented by the following frequency-domain transfer function *H*(*f*):

(1) $$\begin{array}{c} H\left(f\right)=\frac{Y\left(f\right)}{X\left(f\right)}, \end{array}$$

where *f* denotes the frequency (Hz) and *X*(*f*) and *Y*(*f*) represent the Fourier transforms of *x*(*t*) and *y*(*t*), respectively.

Since an IMFT can only measure *y*(*t*), to ensure that the actual GRF signal *x*(*t*) can be approximated closely by *y*(*t*), conventional IMFTs were designed to behave like a distortionless transmission system in the low-frequency range. An SISO system is a distortionless transmission system if it satisfies the following condition:

(2) $$\begin{array}{c} y\left(t\right)=kx\left(t-t_{d}\right), \end{array}$$

where *t* is the time variable, *k* is an arbitrary constant, and *t*_*d*_ is the time delay of this distortionless transmission system. Therefore, the transmission is considered to be distortionless if the input and the output have identical wave shapes with a proportionality constant *k*. A delayed output that retains the input waveform is also considered distortionless. These specifications of distortionless transmission can be converted into the frequency domain by taking the Fourier transform of (2) which yields

(3) $$\begin{array}{c} Y\left(f\right)=kX\left(f\right)e^{-j2\pi {ft}_{d}}. \end{array}$$

Therefore, the corresponding amplitude response is

(4) $$\begin{array}{c} \left|H\left(f\right)\right|=k, \end{array}$$

and the phase response is

(5) $$\begin{array}{c} ∠H\left(f\right)=-t_{d}2\pi f. \end{array}$$

Hence, a distortionless transmission system must have a constant amplitude response and a phase response that declines linearly with frequency *f*. By modeling the GRF transmission dynamics of an IMFT as a linear time-invariant second-order system with natural frequency *f*_*n*_ and a damping ratio *ξ*, its amplitude and phase responses can be expressed, respectively, as [50]

(6) $$\begin{array}{c} \left|H\left(u\right)\right|=\frac{1}{\sqrt{{\left(1-u^{2}\right)}^{2}+4\xi^{2}u^{2}}} \end{array}$$

and

(7) $$\begin{array}{c} ∠H\left(u\right)=-\tan^{-1}\left(\frac{2\xi u}{1-u^{2}}\right), \end{array}$$

where the dimensionless frequency variable *u* = *f*/*f*_*n*_. If *f* is much smaller than *f*_*n*_, the amplitude and phase responses of this standard second-order system can be approximated by

(8) $$\begin{array}{c} \left|H\left(u\right)\right|\approx 1 \end{array}$$

and

(9) $$\begin{array}{c} ∠H\left(u\right)\approx -2\xi u. \end{array}$$

Therefore, a linear time-invariant second-order system behaves like a distortionless transmission system when *f* < <*f*_*n*_. This is the reason why the structural natural frequency of a conventional IMFT needs to be considerably higher than the bandwidth of the GRF signal.

Experimental studies have found that, on average, 99% of the vertical direction GRF signal power was contained under 12.75 Hz when walking at a comfortable speed [12]. Nevertheless, human GRF contain frequency components as high as 60 Hz for walking [51] and 100 Hz for running [52]. To quantitatively demonstrate the importance of high structural natural frequency of the treadmill structure, we assume that the natural frequency *f*_*n*_ to be 45 Hz (Kram et al. [45] indicated that the vertical direction structural natural frequencies of the six force treadmills that they reviewed are all lower than 45 Hz). With *f* = 12.75 Hz, the corresponding dimensionless frequency *u* is 12.75/45 ≈ 0.283. By using (6) with *u* = 0.283 and *ξ* = 0, it can be shown that |*H*(*u*)| ≈ 1.087 which represents an 8.7% deviation from the desired specification of |*H*(*u*)| = 1. To reduce such a deviation, two more recently developed IMFTs increase their structural natural frequencies to 160 Hz [45] and 219 Hz [46], respectively. At *f* = 12.75 Hz, the corresponding |*H*(*u*)| improves to 1.006 and 1.003, respectively.

When modeled as a linear time-invariant second order system, it is well known that the structural natural frequency *f*_*n*_ of the treadmill can be determined from

(10) $$\begin{array}{c} f_{n}=\frac{1}{2\pi }\sqrt{\frac{k}{m}}, \end{array}$$

where *k* (N/m) is the stiffness and *m* (kg) is the mass of the treadmill. Clearly, *f*_*n*_ can be increased by reducing the weight of the treadmill. This is the reason why previous force treadmills often removed parts such as side handrails, front rails, and the control panel to make the treadmill lighter. However, these changes also degraded the functionality and safety of the treadmill system. The natural frequency *f*_*n*_ can also be increased by using higher strength materials to increase the stiffness. The lightweight and high strength material requirements inevitably increase the cost of the treadmill.

To relax the high natural frequency requirement for the IMFT structure, this study tries to compensate for the effect of the GRF transmission dynamics of the treadmill by identifying its transfer function model. In particular, by applying an excitation force *x*(*t*) to the treadmill track surface and measuring the resulting *x*(*t*) and *y*(*t*), we can identify the transfer function from *x*(*t*) to *y*(*t*) from (1). Using the inverse dynamic model of the identified transfer function, we can then estimate the actual GRF from

(11) $$\begin{array}{c} x_{c}\left(t\right)=F^{-1}\left\{{\hat{H}}^{-1}\left(f\right)Y\left(f\right)\right\}, \end{array}$$

where $\hat{H}\left(f\right)$ represents the identified transfer function of the treadmill GRF transmission dynamics. In the remaining parts of the manuscript, *x*(*t*), *y*(*t*), and *x*_*c*_(*t*) will be referred to as the actual, the uncompensated, and the compensated GRF signals, respectively. The experimental procedure for implementing the proposed approach will be described in the following subsection.

### 2.2. The Experimental Procedure

**Figure 1:**
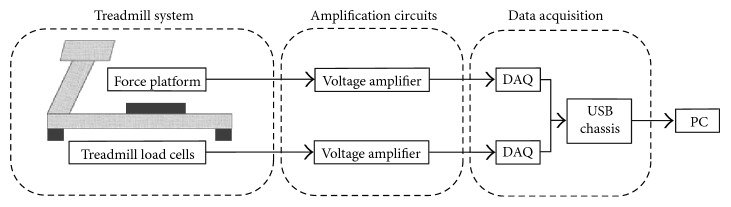
Configuration of the experimental system.

**Figure 2:**
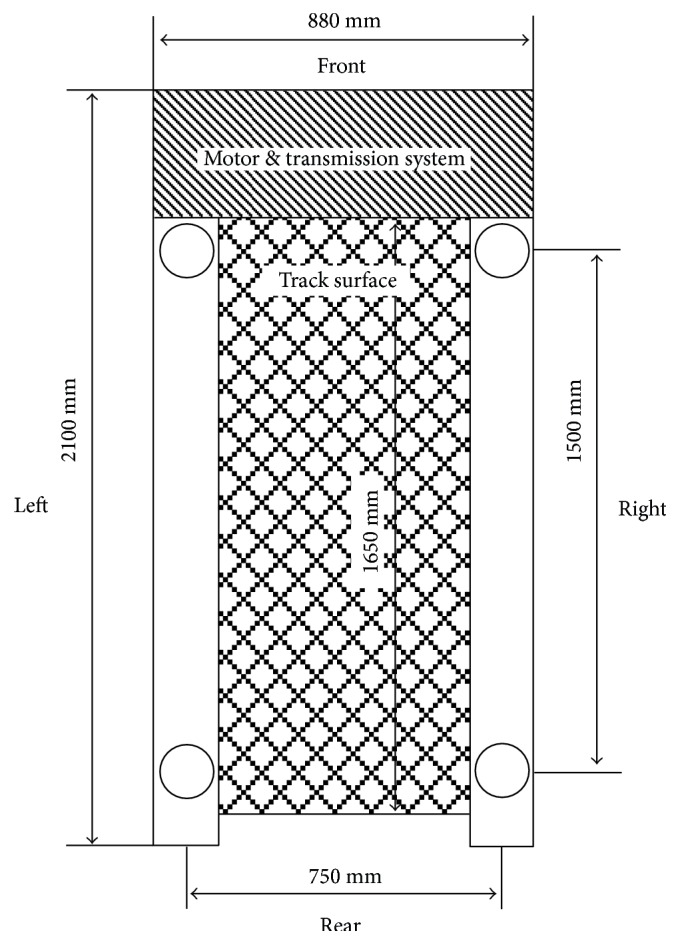
Top view of the treadmill surface.

Figure 1 illustrates the configuration of the experimental system which consists of two subsystems, namely, a force treadmill and a force platform. The treadmill (7355, Fit Plus, Taiwan) has bed dimensions of 1.5 m length and 70 cm width. The speed control system provides a range from 0 to 22 km/hr with a minimum increment of 0.1 km/hr. The weight of the treadmill is 150 kg. To convert this standard exercise treadmill into a force treadmill, this study installed four load cells (Sensolink SLP-1 with maximum capacity of 100 kg) into the legs that support the treadmill body. The four circles shown in Figure 2 specify the location of the force transducers.

As shown in Figure 1, to measure the actual GRF signal *x*(*t*), a force platform is placed at the center of the treadmill track surface. Similar to a commercially available force platform, the force platform built here is a rectangular plate with force transducers located at its four corners. The force treadmill and the force platform employed in this study use the same load cell unit. The size of the platform is 40 cm by 40 cm. We have carefully compared the measurements obtained by this force platform and a commercial force platform (Kistler 9286AA) to verify comparable repeatability and accuracy.

After amplification, analog voltage signals obtained by the four load cells of the force treadmill are converted to digital signals via a four channels, 24 bit DAQ (data acquisition) card (NI 9234). The voltages generated by the load cells of the force platform are also processed by an independent but identical set of voltage amplifiers and a DAQ card. The digitized force signals were sent to a PC using a USB chassis (NI cDAQ-9174) and low pass filtered by a distortionless phase 20th-order Butterworth filter with a cutoff frequency of 150 Hz. The sampling frequency was set to 1024 Hz. The experimental system used the graphical programming environment NI LabVIEW (National Instrument, Austin, TX, USA) for performing system control, signal processing, and graphical user interface (GUI) functions.

The experimental work consists of two phases. The first phase identifies the GRF transmission dynamics of the treadmill by finding its transfer function model. A dead blow hammer with a nonmarring head was used to strike the center of the force platform. By measuring the resulting *x*(*t*) and *y*(*t*) with the force platform and force treadmill, respectively, the transfer function of the GRF transmission dynamics was determined from (1). The second phase of the experimental work was to assess the accuracy of the estimated GRF signals. The test input signals were produced by asking ten male subjects (age 24.20 ± 3.29 years, weight 73.09 ± 15.42 N) to walk “on the spot” for 20 seconds when standing on the force platform which was placed on the center of the treadmill track surface. The fidelity of the estimated GRF was evaluated quantitatively by its total harmonic distortion (THD), defined as

(12) THD=∫0Tx^t−xt2dt∫0Tx2tdt,

where $\hat{x}\left(t\right)$and *x*(*t*) represent the estimated and actual GRF signals, respectively. In this study, the duration for each of the walk-on-the-spot tests was *T* = 20 s. Note that, by defining the distorted signal as the difference between $\hat{x}\left(t\right)$ and *x*(*t*), the THD represents the ratio of the energy of the GRF estimation error signal to the energy of the actual GRF signal.

## 3. Results and Discussions

**Figure 3:**
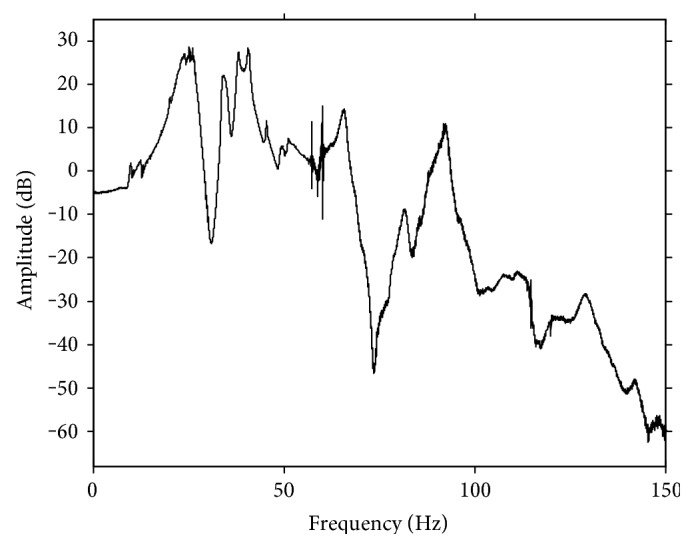
The amplitude spectrum of the identified transfer function.

Figure 3 plots the amplitude spectrum of the identified transfer function of the treadmill GRF transmission dynamics obtained in the first phase of the experimental study. As shown in Figure 3, the amplitude response of the identified transfer function is very different from that of a distortionless transmission system. Specifically, its amplitude response is relatively flat only in the low-frequency range of 0 to 5 Hz and becomes highly oscillatory in the higher frequency region. This clearly reveals the importance of compensating for the effect of GRF transmission dynamics to improve GRF measurement accuracy for an IMFT.

**Figure 4:**
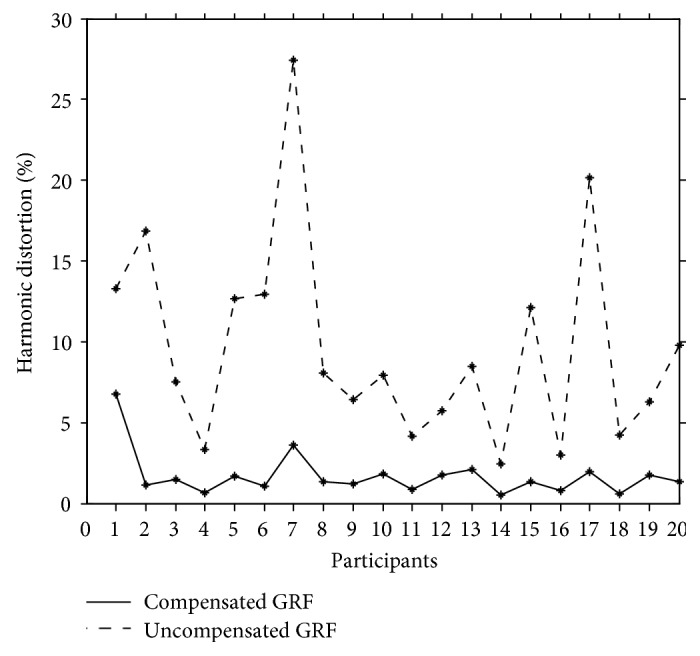
Total harmonic distortions of the estimated GRF signals for the walk-on-the-spot experiment.

**Figure 5:**
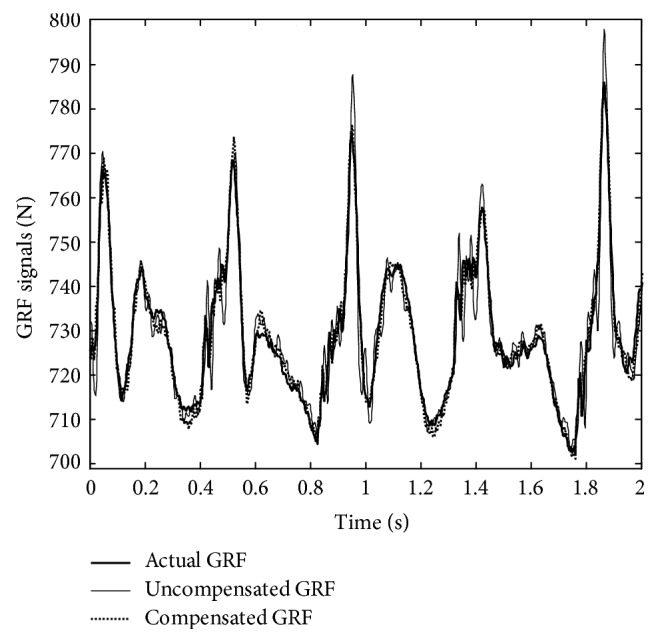
GRF signal time responses of a typical 2-second period for the walk-on-the-spot experiment.

To demonstrate the efficacy of the proposed approach, based on the data obtained in the second phase of the experimental study, the THDs were computed by using the uncompensated and the compensated GRF signals as the estimated GRF signal. The resulting THDs for the 20 participants of the walk-on-the-spot experiment are plotted in Figure 4. As shown in Figure 4, the THDs obtained by the compensated GRF are considerably smaller than the THDs of the uncompensated GRF. In particular, for the uncompensated GRF, the mean and standard deviation of the THDs are 9.64% and 6.3%, respectively. In comparison, by using the compensated GRF as the estimated GRF, the proposed approach reduces the mean of the THDs to 1.69% and the standard deviation of the THDs to 1.38%. Such improvements can also be observed from Figure 5 that plots the time responses of the actual, the compensated, and the uncompensated GRF signals for a typical 2 s period of the walk-on-the-spot experiment. As shown by Figure 5, the time responses of the actual and compensated GRF signals are relatively close. In contrast, the uncompensated GRF signal tends to oscillate around the actual GRF signal and often overshoots the actual GRF signal, particularly at the sharp corners of the actual GRF time response profile.

**Figure 6:**
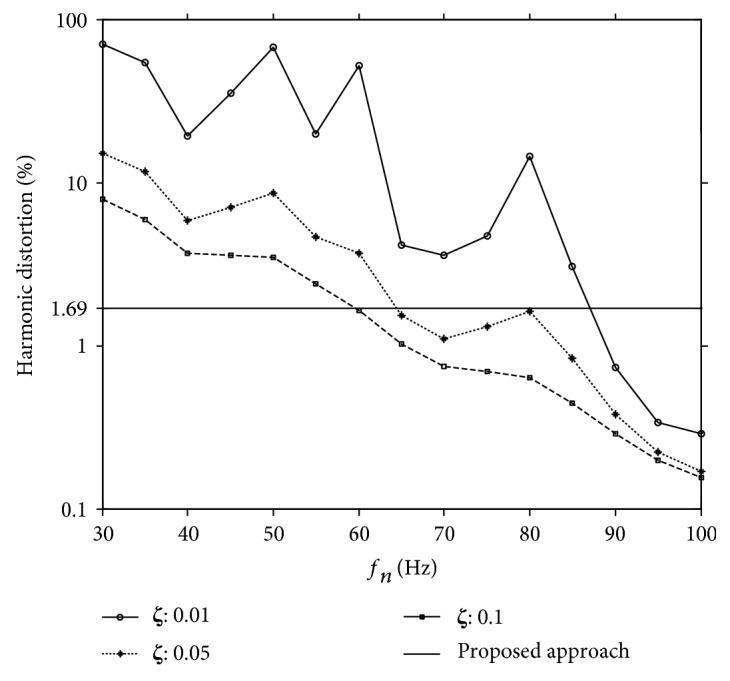
The total harmonic distortion of a second-order IMFT model for the walk-on-the-spot experiment.

To compare the efficacy of the proposed approach to the conventional IMFT design, the IMFT was modeled as a second-order linear system whose frequency spectra can be represented by (6) and (7). With the actual GRF signal of the walk-on-the-spot experiment as the input and the corresponding output of the second order linear system of (6) and (7) as the estimated GRF, the THDs can be determined for the IMFT mathematical model. The resulting mean THDs of the twenty participants are plotted in Figure 6 as a function of *f*_*n*_ for *ξ* = 0.01, 0.05, and 0.1. As expected, THD decreases with the increasing *f*_*n*_. Since Kram et al. [45] indicated that the vertical direction natural frequencies of the six force treadmills that they reviewed are all lower than 45 Hz, we first inspect the THDs for *ω_n_* = 45 Hz. Based on the results of Figure 6, when *ω_n_* = 45 Hz, the THDs are 7.07%, 3.59%, and 2.07% for *ξ* = 0.05, 0.1, and 0.2, respectively. Note that the solid line of Figure 6 corresponds to the mean THD obtained by the proposed approach which is 1.69%. In order to reduce THD to be smaller than 1.69%, the natural frequency has to increase to 88 Hz, 65 Hz, and 60 Hz for *ξ* = 0.01, 0.05, and 0.1, respectively.

**Figure 7:**
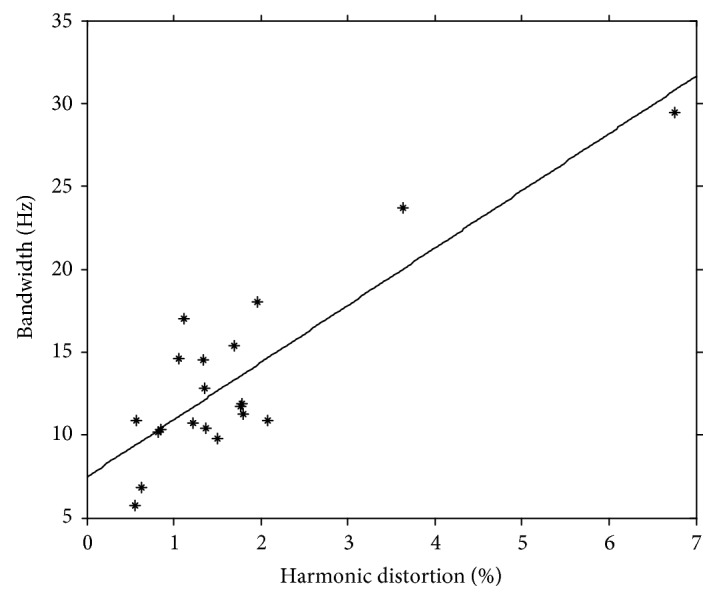
The scatter diagram of the total harmonic distortion and the 98% bandwidth of the compensated GRF.

As shown in Figure 6(), THDs vary from person to person. Although accurate prediction of individually dependent THDs does not seem possible, it is still valuable to understand factors that can influence the accuracy of the estimated GRF. Considering the potential influences of noise on the system identification process in the high-frequency range, it is hypothesized that the THD is positively correlated with the bandwidth of the GRF signals. Due to unavailability of the actual GRF signal during the normal treadmill operations, this study investigates the association between the bandwidth of the compensated GRF signal and its THD. In particular, by specifying the 98% bandwidth as the portion of the signal spectrum in the frequency domain which contains 98% of the signal energy, Figure 7() depicts the scatter diagram of THD versus 98% bandwidth of the compensated GRF signal. With a *p* value of 1.41 × 10^−7^, the value of the corresponding correlation coefficient is 0.891. Such a strong correlation demonstrates that the inaccuracy of the compensated GRF signal increases with its bandwidth. To the best of our knowledge, such an association between GRF frequency content and the GRF measurement accuracy has never been studied systematically. Such knowledge can help us estimate the degree of inaccuracy of the GRF measurements in dealing with GRF signals with different frequency contents.

The experimental results presented in this work demonstrate the feasibility of the proposed approach. However, the success of the approach relies on the linear system assumption of (1). For a poorly constructed treadmill, this assumption of linearity may not be valid. It is also possible that the GRF transmission dynamics of the treadmill are too complex to be compensated accurately. Therefore, choosing a treadmill with a relatively solid structure should be an important consideration in implementing the proposed approach.

Since increasing the structural natural frequency of an IMFT tends to increase its cost and an IMFT with poor rigidity may be too difficult to be compensated accurately, a possible compromise between cost and performance of an IMFT is to build a relatively rigid but inexpensive IMFT with a less than ideal structural natural frequency and then improve its GRF measurement accuracy with the proposed approach. A possible future work is to systematically study the tradeoffs between the cost and accuracy for such a hybrid hardware-software force treadmill design. For the existing IMFTs, the proposed approach can be used to examine the frequency responses of their GRF transmission dynamics. This can help us better understand the dynamic behaviors of the existing IMFTs since their frequency responses have rarely been investigated systematically. The proposed approach can also be applied to quantify the accuracy of the existing IMFTs by computing the distortions of their GRF signals. If necessary, the proposed approach can also be used to improve their GRF measurement accuracy by compensating the effect of the GRF transmission dynamics.

## 4. Conclusion

The goal of this work is to reduce the cost and extend the applicability of force platform-instrumented treadmills (force treadmills). By identifying the influences of treadmill structural dynamics on ground reaction force (GRF) measurements and by installing force transducers underneath the treadmill body, a standard exercise treadmill can be converted to a force treadmill. A previous work showed that treadmill structures need to be highly rigid in order to ensure that the resultant force measured by these force sensors closely approximates the actual GRF applied to the track surface. The high cost for building such treadmills has limited their adoption.

To relax the requirement of high structural rigidity, the proposed approach adopts a system identification approach to model the GRF transmission dynamics from the treadmill track surface to the force sensors underneath the treadmill. By using the inverse dynamic model of the identified transfer function, the approach can be used to estimate the actual GRF by compensating for the effect of the GRF transmission dynamics of the treadmill.

In addition to developing a compensation method to enhance the GRF measurement accuracy, this work introduces an experimental procedure to assess the accuracy of the estimated GRF signals. As shown by the test results obtained from the *walk-on-the-spot* experiment, the mean total harmonic distortion of the estimated GRF signals is only 1.69%. This study also found that the inaccuracy of the estimated GRF signal increases with its bandwidth. In addition to converting standard exercise treadmills to force treadmills, the proposed approach can be used to assess and improve the GRF measurement accuracy of existing force treadmills.
